# Consumption of Health-Related Content on Social Media Among Adolescent Girls: Mixed-Methods Pilot Study

**DOI:** 10.2196/11404

**Published:** 2019-03-01

**Authors:** Miriam P Leary, Emily N Clegg, Madison E Santella, Pamela J Murray, Julie S Downs, Melissa D Olfert

**Affiliations:** 1 Division of Animal and Nutritional Sciences Davis College of Agriculture, Natural Resources, and Design West Virginia University Morgantown, WV United States; 2 School of Medicine West Virginia University Morgantown, WV United States; 3 Social and Decision Sciences Dietrich College of Humanities and Social Sciences Carnegie Mellon University Pittsburgh, PA United States

**Keywords:** adolescent, female, social media, health information, health behaviors

## Abstract

**Background:**

Consumption of health- and fitness-related social media content is a predominant behavior among teenage girls, which puts them at risk for consuming unreliable health-related information.

**Objective:**

This mixed-methods study (qualitative and quantitative) assessed health behavior attitudes and practices as well as social media use among adolescent girls. Additionally, similar practices and behaviors of adults who regularly interact with this population were studied.

**Methods:**

Girls aged 12-18 years were recruited to complete a 28-item survey and participate in a 45- to 60-minute focus group. Adults who regularly interact with adolescent girls, including parents, teachers, and healthcare professionals, were recruited from the local community and given a link to provide online consent and complete a survey.

**Results:**

A total of 27 adolescent girls participated in one of nine focus groups. Participants included 18 high school (age: mean 16.1 years; SD 1.3 years) and 9 middle school (age: mean 12.4 years; SD 0.7 years) girls. Eleven adults completed the online survey. Adolescents used social media to communicate and connect with friends, rather than as a source of health information. Although adolescents may see health-related content, most do not follow health-related pages or share such pages themselves, and fewer are actively searching for this information. Adolescents tend to trust information from familiar sources, and the participants reported that they do not follow official news accounts. Adults considered modeling and discussing healthy behaviors important and reportedly expected adolescents to see some level of health-related, especially fitness-related, content on social media.

**Conclusions:**

Education interventions are warranted for both adolescents and adults with whom adolescent girls regularly interact, in the areas of sedentary behavior to guide them to access reliable online health-related information and be judicious consumers of online health information.

## Introduction

More than one in five adolescents in the United States are obese [1], which could be attributed, at least in part, to unhealthy lifestyle behaviors such as inadequate physical activity and a poor diet. Only 17% of high school girls report levels of physical activity that meet aerobic guidelines; unfortunately, these levels are lower than those of their male counterparts [2]. Further, high school girls consume high amounts of sugar, practice fad dieting, and demonstrate weak nutritional knowledge and unhealthful eating habits [3]. The transformative period of adolescence is an ideal time for individuals to begin adopting positive health behaviors, especially with regard to eating habits and activity. Making positive health-related choices during adolescence can prepare an individual for a lifetime of health and wellness. Conversely, if poor eating habits and sedentary behavior are adopted during adolescence and practiced over decades, significant health-related consequences could occur [4].

Public health messages have increasingly targeted children and adolescents, but mass media efforts that have previously proved effective (ie, newspapers and television) may no longer be culturally relevant in today’s society. Most (92%) adolescents search health-related information online [5], but far less (10%) adolescents reference more dated sources such as books, television news (9%), and newspapers (3%) [6]. In fact, one focus group study indicated that adolescents search for a wide range of health topics on the internet, including nutrition [7]. Public health and health care-related practices and research targeting adolescents must recognize that teenagers are at the forefront of the transition from traditional to electronic media, especially social media. Indeed, 71% of adolescents report using more than one social media site [8], and 45% of adolescents report using social media sites every day [9].

Adolescent girls appear to be at greater risk of consuming unreliable health-related information on social media than their male counterparts [6]. Age, gender, race, physical activity level, and overall health are significant predictors of the amount of information adolescents report receiving from social media sites [6]. People who are older, female, more active, and in better health are more likely to use social media sites for health information [6]. Further, health- and fitness-related social media content is predominantly consumed (liked or followed) by teenage girls [10].

Clearly, research investigating the role of social media in adolescent girls’ health behaviors is warranted; however, the majority of studies investigating adolescent use of social media focuses on the impact on their psychological well-being. These studies have found unfavorable effects of social media on mental health including negative mood self-objectification, body dissatisfaction, social comparison, eating behaviors, decreased self-esteem, weight dissatisfaction, drive for thinness, and peer competition [11-19]. Even “fitspiration” images and posts that aim to inspire people to live healthy and fit lifestyles reinforce the overvaluation of physical appearance, eating concerns, and excessive exercise that could have serious negative implications on adolescents’ psychological and emotional health [20]. Despite these findings, the role of social media on influencing health behavior practices in adolescent girls is unclear.

Adolescents have access to a variety of health information via social media sites [8]. Most adolescents do not turn to social media sites for health-related information, but this should not be generalized for the whole population [6]. One in ten adolescents reported that they get “a lot” of health information from social media sites and 23% said they get at least “some” information from such sites. Some adolescents simply come across health information through links on social media sites (6%) [6]. In general, adolescents tend to be wary of posting health-related questions or viewing health-related information on social media, especially when their names can be associated with such information [6,21].

Many of these findings are from representative population surveys, but the relationship between the use of social media by adolescent girls and its effects on health behavior is likely complex and multifactorial. Understanding these relationships requires more qualitative formative research. Therefore, this mixed-methods pilot research study examined the use of and exposure to social media among adolescent girls with regard to health-related content, with a goal of identifying potential avenues for targeting their use of social media and its effects on health behavior. These findings will inform the creation of materials aimed at increasing safe consumer practices of social media use among this population.

## Methods

### Design

This pilot study assessed adolescent girls’ attitudes, practices, and social media use related to health behavior as well as practices and behaviors of adults who regularly interact with them, by using qualitative and quantitative methods. This study was approved by the Institutional Review Board of West Virginia University (#1711839385) and Carnegie Mellon University (STUDY2017_00000559). Parents or guardians of the participants gave written informed consent and participants gave informed assent.

### Participants

Adolescent girls aged 12-18 years from local middle schools and high schools were invited to participate in 45- to 60-minute focus groups and complete an anonymous survey. Recruitment emails were sent to local principals and school administrators and personnel asking them to invite girls aged 12-18 years to participate in the study. Principals and administrators who agreed were provided an email to be sent to students and their parents. Adolescent participants were compensated with US $25. Independently, we recruited adults from the same schools where adolescents were recruited, who regularly interacted with adolescent girls, including parents, teachers, and health care professionals, and provided them a link to an online consent form and a 10-minute anonymous survey. Adults were not compensated for participating in the online survey. All those interested in completing the survey had the option to do so after providing consent. Adults and adolescent participants were not necessarily related. There was no bias against ethnicity or race.

### Instruments

Adolescent girls completed a 28-item Qualtrics survey, which collected demographic information (eg, age, year in school, height, and weight) and other responses detailed below, and then participated in a focus group discussion. Adults completed a 13-item Qualtrics survey described below. Numerical response scores were used for quantitative analysis. The questions used for these surveys were not derived from validated surveys.

#### Adolescent Survey

To investigate the levels of physical activity, participants were asked whether they have physical education (PE) class (score: 1=yes and 2=no) and how much activity was done outside of the PE class (score: 1=every day to 5=never). To investigate sedentary behavior, the survey inquired if participants felt that the word “sedentary” described them (score: 1=describes me extremely well to 5=does not describe me). Participants also estimated the hours spent in sedentary activities such as sitting or not moving and were asked to attribute reasons for periods of no activity (boredom, laziness, fatigue, time limitations, physical limitations, financial limitations, activities enjoyed are sedentary, and other). Six questions assessed hours spent on social media, device used (computer, tablet, or cell phone), and timing (day and weekend). The questions used for this survey were not derived from a validated survey.

#### Adult Survey

The survey investigated health behaviors, efforts to model healthy behaviors and have health-related conversations with adolescents, use of social media, and perceptions of adolescents’ use of social media. Adult survey questions were independent of adolescent survey questions. The questions used for this survey were not derived from a validated survey.

### Focus Groups

Trained female researchers moderated focus groups and took notes. A semistructured protocol following standard focus group guidelines [22] was administered by trained researchers [22,23]. Focus group participants were asked questions designed to explore their attitudes toward social media-related topics, identify physical activity and sedentary behaviors, and discover how they use technology and social media. A trained note taker made comprehensive notes on a laptop computer at each focus group, and the proceedings were digitally audio recorded. Within 48 hours of the end of each focus group, a second note taker transcribed the digital recording. These notes were reviewed by the focus group moderator for clarity, thoroughness, and accuracy.

### Analysis

Descriptive statistics were used to summarize numerical questionnaire data with Microsoft Excel (Microsoft Corp, Redmond, WA) and were reported as mean (SD). Content analysis to identify common themes was conducted by two independent researchers (authors MPL and MES) with a tiebreaker (author MDO) when necessary. The researchers discussed independent content analysis findings, and data were constantly analyzed and compared to determine saturation of repetitive concepts (ie, point at which no new information, trends, or themes emerge from the data) [24].

## Results

### Participants

A total of 27 adolescent girls participated in one of nine focus groups (4 middle school groups and 5 high school groups) consisting of 2-5 girls each, including 18 high school (age: 16.1 [SD 1.3] years) and 9 middle school (age: 12.4 [SD 0.7] years) participants. Eleven adults completed the online survey.

### Adolescent Participant Survey

Responses of the adolescent participant survey are presented in Table 1. High school participants reported spending an average of 3 hours and 23 minutes daily on social media. Most (94%) accessed social media on their phone. They reported accessing social media mostly late at night during weekdays and during the day on weekends. In addition, they used Instagram (89%), SnapChat (89%), YouTube (74%), and Pinterest (61%), with most social media time related to Instagram and SnapChat. Half of them reported participating in a PE class, and almost all (94%) reported undertaking physical activity outside of PE more than twice a week. Periods of inactivity were attributed to boredom, tiredness, or laziness (56%, 78%, and 39% of respondents, respectively).

Middle school participants reported spending an average of 1 hour and 14 minutes daily on social media on both their phone and computer. They reported accessing social media mostly after school on weekdays and during the day on weekends. In addition, they used YouTube (100%), Instagram (70%), and Snap Chat (50%), with most social media time related to YouTube (32%) and Instagram (30%). One subject did not respond to physical activity-related survey questions. Of those who responded, all reported participating in a PE class and almost all reported participating in physical activity outside of the PE class at least twice a week. Periods of inactivity were attributed to tiredness, boredom, and physical limitations (50%, 20%, and 20% of respondents, respectively).

### Adolescent Participant Focus Groups

#### Adolescent Participants’ Self-Described Use of Social Media

Snapchat and Instagram provide social connections for high school participants; one participants said, “it’s nice to see other people and what’s happening in their lives.” Each provided a different type of opportunity: “Snapchat is for funny posts, Instagram is where you try and look good - your best life.” Although most participants had Twitter accounts, they did not use it as much. Facebook was not used and considered “out of date” and “a platform for older people.” Many girls used YouTube and had a Pinterest account, but did not report using either frequently. Middle school girls also reported using similar platforms, with more time spent on YouTube because “it’s fun to waste time on.” Middle school participants also stated that Facebook is more common among older generations; one participant commented, “my grandma has Facebook.”

**Table 1:** Adolescent participant survey responses.

Question and response choices		High school (N=18)	Middle school (N=9)
Age (years), mean (SD)		16.1 (1.3)	12.4 (0.7)
**Grade, n (%)**			
	6th	N/A^a^	4 (44.4)
	7th	N/A	4 (44.4)
	8th	N/A	1 (11.1)
	9th	3 (16.7)	N/A
	10th	5 (27.8)	N/A
	11th	6 (33.3)	N/A
	12th	4 (22.2)	N/A
**Do you have PE^b^** **class? n (%)**			
	Yes	9 (50)	8 (88.9)
	No	9 (50)	0 (0)
**Do you participate in physical activity outside of PE class? n (%)**			
	Everyday	8 (44.4)	2 (22.2)
	4 or more times	4 (22.2)	3 (33.3)
	2-3 times	5 (27.8)	2 (22.2)
	Less than 2 times	1 (5.6)	1 (11.1)
	Never	0 (0)	0 (0)
**How well does “sedentary” describe you? n (%)**			
	Extremely	0 (0)	0 (0)
	Very well	0 (0)	0 (0)
	Moderately	6 (33.3)	0 (0)
	Slightly	6 (33.3)	4 (44.4)
	Does not describe me	6 (33.3)	4 (44.4)
**Periods of inactivity attributed to... n (%)**			
	Boredom	10 (55.6)	2 (22.2)
	Laziness	7 (38.9)	1 (11.1)
	Too tired	14 (77.8)	5 (55.6)
	No time to be active	4 (22.2)	1 (11.1)
	Physical limitations	2 (11.1)	2 (22.2)
	Financial limitations	0 (0)	0 (0)
	Activities I enjoy are sedentary	6 (33.3)	7 (77.8)
	Others (school/homework)	5 (27.8)	3 (33.3)
Time spent on social media, mean (SD)		3 h 23 min (0.11 h)	1 h 14 min (0.03 h)
**How do you access social media? n (%)**			
	Cell phone	17 (94.4)	8 (88.9)
	Computer	9 (50)	8 (88.9)
	Tablet	3 (16.7)	6 (66.7)
**Time spent on social media**			
	Weekdays	Late night	After school
	Weekends	During the day	During the day
**Which apps do you use? n (%)**			
	Instagram	16 (88.9)	7 (77.8)
	SnapChat	16 (88.9)	5 (55.6)
	YouTube	14 (77.8)	9 (100)
	Pinterest	11 (61.1)	6 (66.7)
	Twitter	8 (44.4)	0 (0)
	Google+	3 (16.7)	3 (33.3)
	Buzzfeed	2 (11.1)	2 (22.2)
	Tumblr	2 (11.1)	1 (11.1)
**Time spent on each (%)**			
	SnapChat	43	20
	Instagram	36	30
	YouTube	24	32
	Twitter	10	0
	Pinterest	6	12.5
	Facebook	3	0

^a^N/A: not applicable.

^b^PE: physical education.

For both middle and high school girls, all social media was used for communicating and connecting—“to check up on people”—primarily friends, often instead of texting. One individual explained that social media was used to “post for everyone rather than text all of my friends.” Similar social media apps were used among groups of friends, with little differences between participants in the same social circles:

I feel like we use Instagram and Snapchat more because most people have those. If I got Twitter or something, I wouldn't be on it as much and it wouldn’t be as interesting, because not all of my friends would be on it to interact with and see what they are doing.

Participants used social media because they felt that “if I don’t get on for a few days then I will miss out on jokes” and they would “feel behind.” Social media also allows participants to connect with a larger online audience, including strangers, as a way to convey updates about their lives. Specifically, adolescents reported using Instagram “just for fun” and to connect with people, while Snapchat was considered more personal; for example, one participant commented that “Snapchat is more personal than texting because you can see your friends’ faces.” Participants were motivated to use Snapchat by “maintaining streaks,” or sending Snapchats back and forth for consecutive days without breaking the chain of communication, which creates a record that becomes important to keep. Other forms of social media were used as entertainment (eg, “Pinterest draw you in with crafts and stuff”) to access what is going on in the world (eg, “Twitter is to see the bigger world”) and as a source of information, but this is often in relation to what their friends or celebrities are doing. This group primarily followed friends on social media, but also followed celebrities, athletes, and health-related pages, and they “find people on my own or hear about from friends who to follow.”

Posts seen on social media by high school girls are typically life updates from friends sharing what they are doing, pop culture, or current events from well-known figures, including the President or celebrities. Snapchats are sent back and forth as a form of conversation. Posts seen on social media include memes/funny images, advertisements, and posts from celebrities. Occasionally, high school girls see information regarding current events and advertisements, while many middle school girls see posts about sports they are involved in. A few participants mentioned that social media posts are often fake news, music related, or videos. High school participants reported posting on social media to share funny posts, like memes, or a life update that shows or describes what they are doing or who they are with and may include a picture or selfie. Participants also shared about events, including sports events and life updates (eg, achievements or activities they engage in).

Many participants admitted to not following official news sources. Therefore, regarding the reliability of social media content, participants are more likely to trust information on social media if the source is a verified account, if they have seen it posted repeatedly, or if it is posted by someone they know personally:

I'd definitely trust my friends more than celebrities although I do aspire and like them

They did not trust the information if the post was paid/sponsored or if they saw contradicting information:

Maybe I saw something that was happening and later I found out from the news that it was wrong

Participants recommend researching independently, asking others, or fact-checking to validate untrusted information.

#### Adolescent Participants’ Exposure to Health-Related Information on Social Media

Many participants reported seeing health or nutrition-related posts from friends or official accounts including recipes, pictures of food, or workout posts. Most reported that they did not follow health or nutrition accounts on social media, but that they saw advertisements related to fitness, weight loss, and supplements that they knew were unreasonable:

I usually ignore the ads. They say stuff about “take this pill and make your body look better.”

“Ten foods that can kill you” I didn't believe it.

For participants who followed health-related accounts or people on social media, many of the posts were for healthy recipes or fitness-related content. Further, some participants admitted to searching for health-related content, including workouts and healthy recipes. Both groups reported infrequent posting of health- or nutrition-related information on social media. If such a post was made, it typically related to sports performance or sharing pictures of food that looks good, is homemade, or is from a cool place. Activity-related searches were performed by high school participants to find information on healthy nutrition and workouts. Some participants searched for sports-specific workouts or exercises, conditioning programs, stretches, or drills. It was briefly mentioned that sometimes foods or recipes are investigated to determine their healthiness.

#### Adolescent Participants’ Self-Reported Lifestyle Behaviors

Participants believed that activity is important for a healthy lifestyle. Specifically, it is important for physical and mental health, as “it’s not just the foods you eat, even if you eat super healthy, you have to keep your body in shape to keep your muscles strong.” Physical activity was said to be important to allow optimal performance in sports and because it makes you feel good. Many engaged in physical activity through sports (eg, “sports are a fun and good way to stay active”), working out on their own, and general physical activities like walking dogs and doing chores like yard work. Younger girls report spending their leisure time “playing outside.” Many related activity to maintaining a slim figure, with some stating that “sports help them lose weight”; this topic provoked some concerning reasons for staying active including guilt (eg, “sitting around makes me feel guilty, so I'll work out”). Some expressed pressure in maintaining a healthy weight for fear of being bullied. Girls recommend finding an enjoyable activity, establishing a routine, and incorporating both exercise and healthy eating. Participants reported not using phones during physical activity, especially during team practices, but when phones were involved, they typically used them for counting steps, tracking time or distance, and playing music. Some participants used apps that prescribe specific workouts or training programs, stating “I don’t usually use a phone unless I’m following a workout.” Although online, some participants took screenshots of a work out or routine to reference later when they were more motivated to be active.

Sedentary behavior seemed to be an unfamiliar topic for this audience, but when defined and explained, participants cited social media (via phone or computer), school work, and television as the primary reasons for sedentary behavior, often doing many things at once; they commented, “I just lay in bed and scroll through Instagram” or “normally, I watch TV while on the phone.” Fewer high school participants than middle school participants reported being sedentary while reading. Some of the younger participants also reported being sedentary while listening to music or playing an instrument. Recommendations to reduce sedentary behavior while still participating in preferred activities included activities such as multitasking (eg, playing with the dog while watching television).

Social media pages and people who emphasize healthy lifestyles and fit bodies can leave participants feeling discouraged and envious, but also sometimes encouraged, inspired, and motivated them. One individual explained, “sometimes I’m like, not really envious, but it pushes me to want that more.” High school girls were aware that many advertisements and posts were photoshopped or manipulated and acknowledged that attaining the same physique is often unrealistic:

Guys with really big muscles, that's not aspirable [sic], but people that are just fit and slim and eat healthy, that's helpful.

These participants felt “it is important for girls their age to hear about body empowerment from celebrities” but also recommended using these individuals as motivation to work out or eat healthy and to “keep pictures of your goals.”

### Adult Survey

The majority of adult respondents were parents and teachers of adolescent girls and reported limiting their own sedentary behaviors, getting regular exercise, and attaining healthy nutrition as at least moderately important. Further, these adults felt that it is very important to model health behaviors for adolescent girls: Most modeled limiting social media, but only about half modeled healthy eating, activity, and limiting sedentary behavior. Adults believed it is important to discuss healthy behaviors with adolescents, and most did so by discussing food choices, portion sizes, daily activity, social media use, and healthy body image. Less than half the adults discussed limiting sedentary behavior. For accessing health-related information, almost all recommend talking to parents and two-thirds answered questions directly or encouraged adolescents to talk to a health care practitioner. Only one-third of adults directed adolescents to reliable online sources. Adults expected adolescents to see some level of health-related, especially fitness-related, content on social media (Table 2).

**Table 2:** Adult survey responses (n=11).

Question and response choice		Value
**Which of the following applies to you? n (%)**		
	Parent/guardian of an adolescent	6 (54.5)
	Parent/HCP^a^	1 (9.1)
	Parent/teacher	2 (18.2)
	Teacher only	2 (18.2)
**How important is it to you that you lead a healthy lifestyle? (0=not important, 10=extremely important), mean (SD)**		
	Regular exercise	5.9 (3.4)
	Healthy nutrition	7.6 (2.0)
	Limit sedentary behavior	6.9 (2.8)
**How many times per week do you exercise for at least 20 minutes? n (%)**		
	Never	6 (54.5)
	1-2 times/week	1 (9.1)
	3-4 times/week	2 (18.2)
	Most days	2 (18.2)
**How active is your job? n (%)**		
	Sedentary	2 (18.2)
	Lightly active	5 (45.5)
	Moderately active	4 (36.4)
About how much time do you think adolescent girls spend on social media each day? mean (SD)		3.4 hours (1.5-5.5)
**How does social media influence? (–5=very negative, 0=no influence, 5=very positive influence), mean (SD)**		
	Healthy eating	0.1 (1.9)
	Activity	–1.4 (2.9)
	Sedentary behavior	-0.25 (3.7)
	Body image	–1 (4.3)
Importance of modeling healthy behaviors (0=not important, 10=extremely important), mean (SD)		9.3 (1.1)
**How do you role model healthy behavior? n (%)**		
	Healthy eating	5 (45.5)
	Limit social media	8 (72.7)
	Healthy activity	6 (54.5)
	Limit sedentary behavior	5 (45.5)
Importance of discussing healthy behaviors with adolescents? (0=not important, 10=extremely important), mean (SD)		9.1 (1.1)
**Which do you discuss with adolescents? n (%)**		
	Food choices	9 (81.8)
	Portion sizes	7 (63.6)
	Daily activity	8 (72.7)
	Sedentary behavior	5 (45.5)
	Social media	10 (90.9)
	Healthy body image	11 (100)
**How do you encourage adolescents to access health-related info? n (%)**		
	Encourage talking to parents	9 (81.8)
	Answer directly	7 (63.6)
	Encourage them to talk to HCPs	7 (63.6)
	Direct to reliable online sources	4 (36.4)
**How much health-related info do you think adolescents see on social? (0=none, 10=a lot), mean (SD)**		
	Nutrition	3.0 (2.5)
	General activity	4.8 (2.4)
	Fitness	6.1 (2.2)
	Sedentary behavior	2.9 (2.1)

^a^HCP: health care provider.

## Discussion

Adolescent girls reported using social media, specifically Snapchat and Instagram, to communicate and connect with friends. These forms of social media are mostly utilized to interact with their peers instead of traditional communication methods such as texting and to share posts that are comical or sports-related or display an aspect of their daily life. Although adolescents may see health-related content, most are not following health-related pages or sharing it themselves, and fewer are actively searching for it; they tend to trust information from familiar sources and do not follow official news accounts. Overall, among both middle and high school girls, social media is used for communicating and connecting with friends, rather than as a source of health information.

Adults influence adolescents’ adoption of healthy habits [25]; therefore, it is critical that adults are aware of the nature of adolescent social media use and encourage healthy use by limiting the time spent on social media. Adequate physical activity, limited sedentary behavior, and proper nutrition are critical health behaviors that, if adopted during adolescence, can contribute to lifelong health [4]. As role models and rule enforcers, parents are critical for promoting adolescents’ healthy habits. Adults in this study indicated that limiting their own sedentary behaviors, getting regular exercise, and attaining healthy nutrition were at least moderately important. However, they felt it was extremely important to model healthy practices and have conversations about health behaviors with adolescent girls. These practices could lay the foundation for establishing habits necessary for a lifetime of healthy behaviors.

Research shows that exposure to social media and “fitspiration” posts lead to more body comparisons and lower self-esteem, specifically in women [11,13,20]. With the increase in the number of social media outlets and time spent on them, the reach of social media is expanding to include younger adolescent girls [10]. In the present study, all adults reported having conversations with adolescent girls about a healthy body image, and in focus groups, the adolescent girls were aware that many advertisements and posts are photoshopped or manipulated and that attaining the same physique is often unrealistic. Paired with the girls’ insistence that adolescents should hear about body empowerment suggests that the conversations adults have with adolescents are effective in promoting a healthy body image.

Adults in this study ranked physical activity as moderately important, and most discuss its importance with adolescent girls. Although only one-third of adults met the physical activity recommendations, almost all adolescents engaged in regular physical activity outside of PE classes. Adults’ efforts to emphasize activity to adolescents appears to be effective in promoting healthy behaviors. However, despite rating the importance of limiting their sedentary behavior higher than that of obtaining regular exercise, more adults discussed the importance of daily activity (73%) than those who discussed limiting sedentary behavior (45%) with adolescent girls.

Although adults considered limiting sedentary behavior important for their own health, less than half of the adults reported having these conversations with adolescent girls. In adolescent surveys and focus groups, the phrase “sedentary behavior” needed to be explained, confirming that this audience is missing out on important health information. Although this population understands the importance of physical activity, interventions and public health messages to limit sedentary behavior are warranted. Obesity is a well-documented outcome of screen media exposure, and in this study, social media use accounted for approximately half of the time adolescent girls spent being sedentary outside school. Importantly, adults’ estimates of adolescent social media use (~3.4 hours of use daily) are in line with adolescents’ self-reported social media use; however, adults do not think social media significantly influences health behaviors. Adolescents may benefit from interventions that replace social media with less sedentary activities, while adults may benefit from information connecting social media use and sedentary behavior with adverse health outcomes, including obesity.

Use of social media gives adolescents access to content about health information of varying degrees of trustworthiness. Girls reported seeing health-, nutrition-, and fitness-related content on social media, often in paid advertisements, and recognized the potential to encounter false information. Although verification (eg, fact-checking) was recommended, adolescent girls relied on less-stringent methods for determining what to trust (eg, posts from people they knew personally). These findings indicate that adolescents are consuming and sharing related information uncritically on social media, which could misinform them about health habits. In addition, they underscore the importance of developing and disseminating materials aimed to increase safe consumer practices of social media use in this audience.

Further, few adults (36%) in this study directed adolescents to reliable online sources of health information. Viewing unverified health-related information on social media, without adult recommendations for accessing reliable online sources, could put adolescent girls at risk for unsafe health practices. Future interventions should aim at providing adults educational materials for reliable, online health information to share with adolescent girls.

One limitation to this pilot study was that the survey used for adolescents and adults was not validated. As described previously, this mixed-methods pilot research study aimed to examine the use of and exposure to social media among adolescent girls with regard to health-related content, with the goal of informing the creation of materials, specifically validated surveys, for increasing safe consumer practices of social media use among this population. We intend to expand this pilot study by developing validated surveys to move this area of research forward. Additional limitations include the use of convenience sampling and failure to acquire ethnic and socioeconomic status data. Further, the present study includes a small sample of adolescents and adults; although the sample was informative for this pilot study and the development of a targeted research study, the generalizability of these findings are limited.

Using a mixed-methods (qualitative and quantitative) approach, this pilot study assessed health behavior attitudes and practices as well as social media use in adolescent girls. Additionally, these practices and behaviors among adults regularly interacting with this population were examined. Although the sample size was small, the data generated are rich and informative, and the focus group data reached the saturation point prior to termination of data collection. Results from the present study offer several potential avenues for targeting the use of social media and its effects on health behavior among adolescent girls, including education interventions for both adolescents and adults with whom these girls regularly interact, in the areas of sedentary behavior to access reliable online health-related information and be judicious consumers of online health information.

The state and use of social media are ever changing, but the potential to use social media as a form of promotion for healthy behaviors, especially among adolescents, will continue to offer promise. Social media campaigns that target this population could provide evidence-based, peer-reviewed information in a culturally relevant and age-appropriate format that could favorably impact adolescents at a transformative time. However, the success of any social media-based intervention will depend on a thorough, comprehensive understanding of the current state of social media use and behavior. Otherwise, any potential benefits of direct social media campaigns are likely to be missed. Therefore, the findings of this pilot study set a broad, informed, and meaningful foundation for any future research aimed at changing or influencing social media and its effects on health behavior.

## Abbreviations

HCP: health care provider
PE: physical education
